# Sensing‐Integrated Patient‐Derived Tumorspheres Predict Chemotherapeutic Efficacy via Local Extracellular pH Dynamics

**DOI:** 10.1002/smll.75149

**Published:** 2026-08-13

**Authors:** Stefania Forciniti, Valentina Onesto, Anna Chiara Siciliano, Helena Iuele, Stefano D'Ugo, Norma Depalma, Cinzia Fasano, Alessandro D'Amuri, Giuseppe Gigli, Marcello G. Spampinato, Loretta L. del Mercato

**Affiliations:** ^1^ Institute of Nanotechnology National Research Council (CNR‐NANOTEC) c/o Campus Ecotekne Lecce Italy; ^2^ Tecnomed Puglia – Technopole for Precision Medicine (Biotech Lecce Hub) c/o Campus Ecotekne Lecce Italy; ^3^ Department of Surgery “Vito Fazzi” Hospital Lecce Italy; ^4^ UOC of Pathological Anatomy “Vito Fazzi” Hospital Lecce Italy; ^5^ Department of Experimental Medicine University of Salento, c/o Campus Ecotekne Lecce Italy

**Keywords:** metabolic pH heterogeneity, pancreatic ductal adenocarcinoma, patient‐derived tumorspheres, personalized drug screening, tumor microenvironment

## Abstract

Pancreatic ductal adenocarcinoma (PDAC) is among the deadliest malignancies, due to late diagnosis, poor therapeutic response, and high inter‐ and intra‐patient heterogeneity. Metabolic alterations, particularly extracellular acidification, critically impair drug response yet remain underexplored in clinical stratification. We present a sensing‐integrated 3D platform that enables non‐invasive time‐resolved mapping of pH dynamics in patient‐derived tumorspheres. Primary PDAC cells from five patients were embedded in an alginate‐based matrix incorporating ratiometric optical pH sensors, allowing real‐time monitoring of local extracellular acidification associated with individual cells during treatment. In contrast to existing bulk or invasive methods, our platform captures extracellular acidification dynamics with high spatial and temporal resolution in a 3D model with potential clinical relevance. Distinct extracellular acidification profiles emerged in response to FOLFIRINOX, gemcitabine, and paclitaxel. These metabolic signatures correlated with treatment efficacy and highlighted patient‐specific drug sensitivities. This platform offers a rapid, scalable tool for personalized drug screening and highlights a potential role of pH metabolic heterogeneity in PDAC chemoresistance. It may complement existing clinical workflows by providing early predictive insights into patient‐specific therapeutic outcomes.

## Graphical abstract

The study introduces a 3D sensing‐integrated platform enabling real‐time, non‐invasive monitoring of pH dynamics in patient‐derived PDAC tumorspheres. It revealed distinct extracellular acidification patterns under FOLFIRINOX, gemcitabine, and paclitaxel, correlating with therapeutic efficacy. Results highlight patient‐specific metabolic signatures, advancing personalized drug screening and offering predictive insights into PDAC chemoresistance and treatment response.

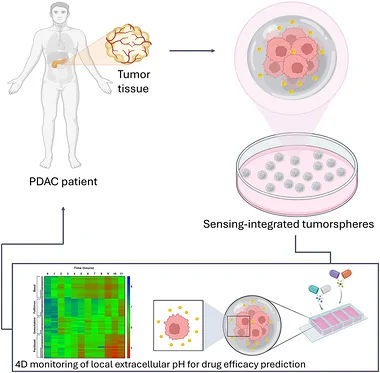

## 1 Introduction

Pancreatic ductal adenocarcinoma (PDAC) is among the most lethal malignancies, with a 5‐year survival rate below 11%, largely restricted to patients eligible for surgical resection [1, 2]. Poor prognosis stems from late diagnosis, limited resectability, and intrinsic resistance to chemotherapy [3, 4, 5]. First‐line regimens such as FOLFIRINOX (5‐fluorouracil, irinotecan, and oxaliplatin) [6] and gemcitabine with or without paclitaxel [7] modestly prolong survival but are frequently limited by severe toxicity [8]. Tumor chemoresistance is further exacerbated by the pronounced heterogeneity of the tumor microenvironment (TME), which in PDAC is characterized by desmoplasia, cellular plasticity, epithelial‐to‐mesenchymal transition, and metabolic rewiring toward aerobic glycolysis with lactate accumulation (Warburg effect) [9, 10, 11, 12]. This metabolic reprogramming generates a highly acidified extracellular milieu that modulates tumor progression and drug efficacy yet remains largely overlooked in clinical decision‐making [13]. Therefore, monitoring extracellular pH fluctuations during chemotherapy can provide functional insights into how TME heterogeneity influences therapeutic responses. Existing methods such as metabolic flux analyzers [14], microelectrodes [15], or Raman spectroscopy [16] are invasive and provide only bulk measurements, whereas imaging modalities such as MRI, PET, and optical techniques (including endoscopy, intraoperative imaging, and fluorescence‐guided surgery) offer higher spatial resolution but remain expensive, technically complex and poorly scalable for routine clinical use [17, 18, 19]. Optical ratiometric fluorescent pH sensors overcome many of these limitations, enabling non‐invasive, real‐time mapping of extracellular pH with high spatial resolution [20, 21, 22, 23]. These dual‐fluorophore sensors use a pH indicator probe (e.g., fluorescein isothiocyanate, FITC) and a reference dye insensitive to pH (e.g., rhodamine B isothiocyanate, RBITC), or intrinsically ratiometric pH indicators (e.g., SNARF, HPTS) to generate concentration‐independent readouts of pH through emission intensity ratios [24, 25, 26, 27]. Traditionally, ratiometric sensors have been embedded in synthetic carriers such as nano‐ and microparticles, electrospun fibers, hydrogels or scaffolds and applied mainly to 2D cultures or immortalized cell lines [28, 29, 30]. However, these conventional models fail to replicate the morphological, molecular and metabolic complexity of PDAC tumors, limiting their predictive power in drug testing [31, 32]. Patient‐derived 3D tumor models more faithfully preserve the native cellular heterogeneity, TME architecture and drug responses, and in vitro drug testing using these models has been shown to reflect antitumor responses in patients, supporting their promise for personalized medicine [33, 34, 35]. Yet, integrating ratiometric pH sensors into these biologically relevant models has remained largely unexplored due to fabrication and optical challenges. Here, we overcome these limitations by establishing a 3D sensing‐integrated platform based on patient‐derived tumorspheres. The term “tumorspheres” refers to an alginate‐based microgel embedding dissociated primary PDAC cells from five patients and ratiometric pH sensors. Alginate was chosen over common matrices such as Matrigel or collagen for its low cost, excellent biocompatibility, optical transparency and negligible autofluorescence suitable for microscopy [36]. Each patient‐derived cell population was phenotypically characterized by flow cytometry to confirm intra‐ and inter‐patient heterogeneity, typical of PDAC [37, 38, 39]. This sensing‐integrated tumorsphere platform enables local extracellular pH mapping associated with individual cells during chemotherapeutic exposure (FOLFIRINOX, gemcitabine and paclitaxel), providing dynamic information on heterogeneity in extracellular acidification patterns and patient‐specific drug sensitivity that would be less evident from bulk measurements alone. By uniting the biological fidelity of patient‐derived 3D models with the quantitative readouts of optical sensing, our approach represents a powerful and clinically scalable strategy to stratify drug responses in the context of metabolic heterogeneity and to advance precision oncology.

## 2 Results and Discussion

### 2.1 Establishment of Patient‐Derived 3D Tumorspheres for Functional pH Sensing and Drug Testing

To establish a robust and clinically relevant 3D platform for functional pH sensing and patient‐tailored drug testing, we generated alginate‐based tumorspheres from primary PDAC specimens (Figure 1). The basic principle involved isolating single‐cell suspension from primary human pancreatic tumor tissues, characterizing cell populations by flow cytometry, and processing them for the generation of tumorspheres. In particular, fresh tumor tissues were collected post‐surgery and processed in a sterile laboratory environment where they were washed in PBS and minced in small pieces. The basis of our experimental procedure was the application of MACS technology for the dissociation of tumor tissue into single‐cell suspension by combining mechanical dissociation with enzymatic degradation of the extracellular matrix. Then, each tumor tissue was enzymatically digested, and the benchtop machine gentleMACS Dissociator was used for the mechanical dissociation steps. After dissociation, samples were filtered in order to eliminate larger, non‐digested particles from the single‐cell suspension. Dissociated cells were subsequently cultured at 37°C in a humidified 5% CO_2_ incubator for about 20–30 days. After expansion, patient‐derived PDAC cells were characterized by flow cytometry to assess epithelial or stromal‐mesenchymal markers expression and to confirm the preservation of phenotypic diversity typical of PDAC. Out of twelve specimens processed, tumorspheres were successfully established from five patients. Importantly, unsuccessful establishment was strongly associated with the histopathological quality of the original specimens; four attempts failed because of insufficient viable cells and extensive necrotic regions, which severely limited the recovery and expansion of viable cells after tissue dissociation; the other three were excluded after histological evaluation revealed a non‐PDAC diagnosis (Figure S1). Patient demographic information and clinicopathological characteristics are shown in Table S1. The study comprised five patients diagnosed with PDAC, including three females and two males, with a median age of 71 years (range, 64–82). Overall, the cohort was relatively homogeneous in terms of clinicopathological features. Tumors were predominantly located in the body–tail of the pancreas (4/5 cases), with a single case arising in the head. Histological classification was consistent across patients, with all tumors identified as ductal adenocarcinoma, including one case with a mucinous component. Tumor grade ranged from G2 to G3, and the majority of patients presented with stage II disease (4/5), while one patient was diagnosed at stage IB. Limited metastatic burden was observed, with only one patient presenting liver metastasis at the time of diagnosis. Histopathological assessment revealed variable tumor cellularity among the analyzed tissue sections, ranging from 60% to 80% tumor cells, with corresponding stromal content between 20% and 40%. Importantly, all samples were processed within a narrow and controlled time window (2–4 h post‐resection), minimizing potential variability related to tissue handling and preservation. For tumorsphere generation, 1 × 10^6^ of dissociated patient‐derived cells were mixed with an alginate matrix and encapsulated using a high‐voltage microdroplet system [21, 23]. Alginate was selected in this study because it provides a highly controllable and reproducible 3D environment, particularly suitable for confocal microscopy and accurate monitoring of extracellular pH dynamics due to its optical transparency, low autofluorescence, and permeability to small molecules, including protons (H^+^). Compared with ECM‐based matrices such as collagen or Matrigel, alginate enables more reliable real‐time pH measurements by minimizing light scattering and background fluorescence. Nevertheless, alginate is biologically inert and lacks cell‐adhesive ligands and only partially captures the biochemical and mechanical features of the desmoplastic stroma, which may lead to a reduced representation of ECM‐dependent processes such as mechanotransduction and matrix remodeling. In the context of this study, this trade‐off was deliberate: our primary aim was to precisely quantify extracellular pH gradients in space and time under well‐defined and reproducible conditions, while maintaining cell viability within a 3D environment. Importantly, despite the inertness of the alginate matrix itself, the patient‐derived tumorspheres retained a heterogeneous multicellular composition, including EpCAM^+^/cytokeratin^+^ epithelial tumor cells, vimentin^+^/N‐cadherin^+^ stromal‐mesenchymal cells, and immune cell populations (Figure 2), confirming that key TME components, including CAFs, are preserved within the 3D model [23, 40].

**FIGURE 1:**
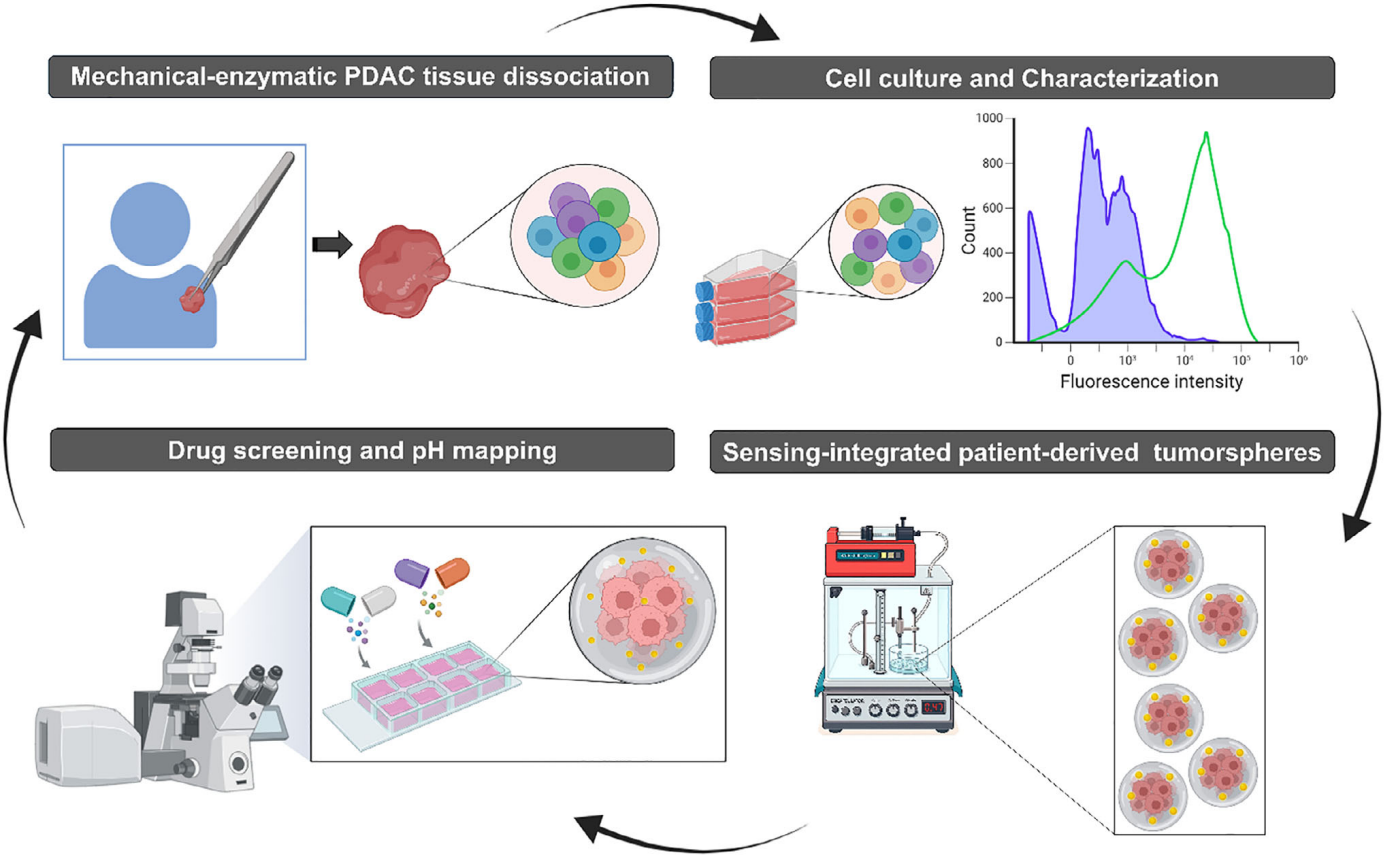
Generation of patient‐derived 3D tumorspheres of PDAC for pH sensing and drug response profiling. Schematic overview of the workflow used to establish alginate‐based tumorspheres from primary pancreatic tumor specimens (*n* = 5). Fresh PDAC tissues were collected post‐surgery and processed to obtain single‐cell suspensions using a combination of enzymatic digestion and mechanical dissociation (MACS Technology and gentleMACS Dissociator, Miltenyi Biotech). Dissociated patient‐derived cells were expanded in culture for ∼30 days, phenotypically characterized by flow cytometry and encapsulated in optically transparent alginate microgels incorporating ratiometric optical pH sensors to generate 3D tumorspheres suitable for extracellular pH mapping and chemotherapeutic testing.

**FIGURE 2:**
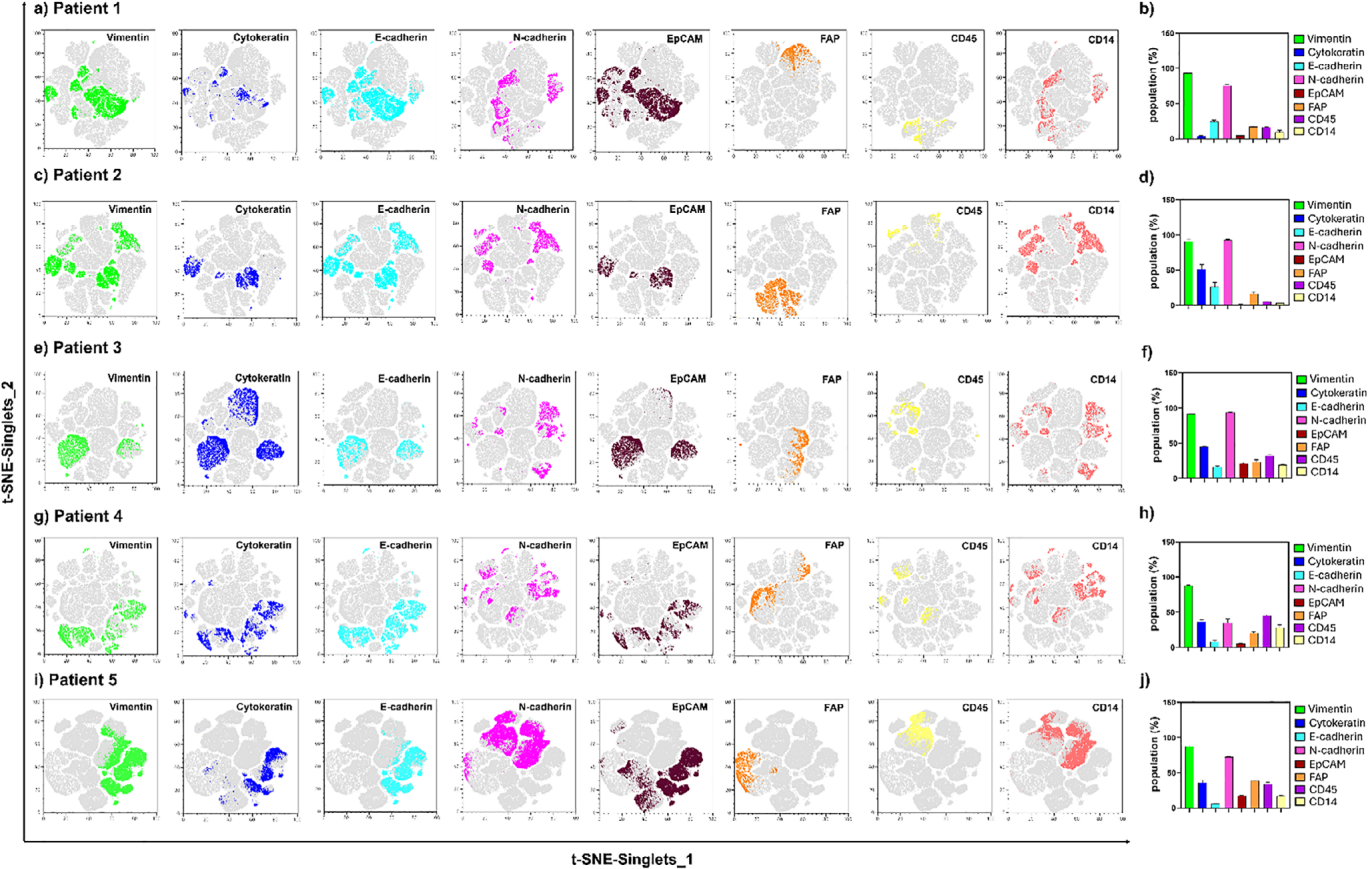
Flow cytometry characterization of PDAC patient‐derived cells. Cells isolated from five PDAC tumor tissues were characterized after culture expansion (subcultured for three passages in culture after thawing). Then, cells were fixed, permeabilized, and stained with fluorescently labeled antibodies for flow cytometry analysis. (a,c,e,g,i) t‐SNE maps showing the spatial distribution of patient‐derived cells expressing mesenchymal‐stromal (Vimentin, N‐cadherin, FAP), epithelial (Cytokeratin, E‐cadherin, and EpCAM), and immune markers (CD45, CD14) in each PDAC patient. Colored points indicate marker‐positive cells, while grey points represent the entire cell population in the dataset. (b,d,f,h,j) Quantitative distribution of epithelial markers, stromal‐mesenchymal markers, and immune markers visualized in t‐SNE maps in each patient‐derived population. Percentages reflect the proportion of cells positive for each marker. Data are presented as the mean ± SEM from triplicate experiments (*n* = 3).

The obtained alginate microgels, produced using the previously established high‐voltage microdroplet platform, were engineered with ratiometric optical pH sensors to enable real‐time extracellular pH monitoring during chemotherapeutic exposure. The structural and functional characterization of this platform, including size distribution, morphology, porous architecture, and diffusion properties, was previously reported in our earlier studies [23, 40].

The microgels had a mean diameter of 151.58 ± 19.30 µm and were generated under fixed encapsulation conditions corresponding to 1 × 10^6^ cells in 110 µL of final encapsulation mixture. Based on spherical geometry, this droplet size corresponds to a theoretical average of approximately 17 ± 6 cells per microgel. This estimate was further supported by confocal z‐stack analysis, which showed an average of 14 ± 4 cells per microgel (mean ± SD). This approach allowed the reproducible formation of uniform 3D tumorspheres that preserve key multicellular components of the TME and patient‐specific heterogeneity, and could be leveraged for dynamic pH sensing and functional drug‐response profiling.

### 2.2 Flow Cytometry Reveals Patient‐Specific Phenotypic Profiles in PDAC‐Derived Cells

PDAC tumors are characterized by extensive intra‐ and inter‐tumor heterogeneity, which is crucial to analyze in detail for understanding the involved biological tumor‐related mechanisms [41]. The hallmark of PDAC is a pronounced desmoplastic stroma enriched in cancer‐associated fibroblasts (CAFs). However, PDAC tumors display distinct histological and cellular compositions that evolve with disease progression [39, 42, 43]. Differences in genetic and molecular features as well as in cell‐type composition have been described, identifying diverse populations of cells within surgically resected PDAC [43]. These comprise altered proportions of ductal cells, fibroblasts, immune cells, especially macrophages and monocytes, and epithelial or mesenchymal cells from early to late PDAC. To determine the cell type identity of patient‐derived cells in the collected PDAC tumor tissues, they were cultured for about 20 days in standard culture conditions (as described in *Methods*). Then, the expression patterns of key markers were analyzed by flow cytometry, providing a quantitative measurement of specific cell surface and intracellular proteins at single‐cell level (Figures S2 and S3). Moreover, to assess whether a prolonged in vitro expansion may potentially introduce cellular selection and phenotypic drift, thereby affecting the preservation of tumor heterogeneity and PDAC subtype representation, the analysis was performed also following freeze–thaw recovery after three passages in culture (Figure 2).

For each patient‐derived cell population, the expression of N‐cadherin and Vimentin, two markers associated with mesenchymal phenotype and upregulated during epithelial‐mesenchymal transition (EMT) process were investigated. Moreover, the epithelial expression profile of primary PDAC cells was obtained by analyzing epithelial‐specific markers E‐cadherin, EpCAM, and Cytokeratin. Finally, to obtain a more comprehensive overview also of the stromal and immune‐related components, the expression of FAP identifying cancer‐associated fibroblasts, CD45 labeling immune cell populations, and CD14 marking cells of the myeloid/monocytic lineage were analyzed (Figure 2).

In Figure 2a,c,e,g,i, the t‐distributed stochastic neighbor embedding (t‐SNE) maps visualized the single‐cell data from the five patients identifying different cell subsets that revealed distinct and heterogeneous expression patterns for the epithelial, mesenchymal, stromal and immune markers analyzed. For each patient, the eight markers mapped onto well‐defined regions of the t‐SNE embedding, highlighting differences in cellular composition and EMT‐related phenotypes. Across all patients, Vimentin expression localized to discrete clusters positioned away from epithelial domains, consistent with the presence of mesenchymal subpopulations. In contrast, Cytokeratin and EpCAM signals were enriched in compact and clearly segregated regions of the embedding, indicating the presence of epithelial tumor cells. Moreover, E‐cadherin displayed a broader distribution across the t‐SNE space, marking epithelial subsets with varying degrees of adhesion properties, whereas N‐cadherin expression appeared more restricted and focal, identifying smaller mesenchymal‐like subsets within each patient. In addition, stromal‐associated cells expressing FAP formed separate clusters, while immune populations expressing CD45 and CD14 occupied distinct regions of the embedding space. Comparative analysis across patients revealed both shared and patient‐specific patterns. Some patients showed a pronounced epithelial signature, with dominant Cytokeratin, E‐cadherin, and EpCAM clusters, whereas others exhibited a stronger mesenchymal‐stromal component, characterized by elevated Vimentin, N‐cadherin or FAP expression. Notably, immune‐related CD45^+^ and CD14^+^ populations were consistently detected across all samples, although their relative abundance varied between patients. The histograms shown in Figure 2b,d,f,h,j summarizing the percentage of cell population positive for the expression of epithelial, stromal‐mesenchymal and immune markers supported these observations, confirming variable proportions of epithelial, mesenchymal, and intermediate phenotypes as well as immune‐associated features across the different patient‐derived cell populations, independently of their spatial context. Importantly, the expression of the analyzed markers remained overall stable across passages and after thawing, without major changes in the relative abundance of the investigated cell populations, although the t‐SNE visualization showed a different spatial organization of marker expression patterns between the first and second phenotypic characterization. These phenomena suggested changes in the multidimensional distribution of cellular phenotypes while preserving overall marker prevalence, mainly due to the stochastic nature of the t‐SNE algorithm and its sensitivity to the multidimensional structure of the dataset. Moreover, fibroblast‐associated populations and, at least to a limited extent, immune‐associated populations were still detectable after expansion with an evident inter‐patient variability in their relative percentages of FAP^+^, CD14^+^, and CD45^+^ cells. It is important to emphasize that the immuno‐stromal composition varies considerably across patients, tumor stage, tumor region, and analytical method. However, the overall ranges of these populations in our samples were consistent with those reported in the literature for PDAC tumors [44, 45, 46, 47].

Taken together, this distinct expression profile for each patient is in accordance with the high intra‐ and inter‐heterogeneity typical of PDAC and could be correlated with an EMT cell state and then, with a more aggressive tumor phenotype, metastatic potential, and poor prognosis.

### 2.3 Physicochemical Characterization of Optical Ratiometric pH Sensors

To develop 3D sensing‐integrated PDAC models, silica‐based ratiometric pH sensors were synthetized as described in Methods. For this purpose, fluorescein isothiocyanate (FITC) and rhodamine B isothiocyanate (RBITC) were selected as pH indicator probe and reference dye, respectively, to produce fluorescent microparticles for ratiometric pH sensing. SEM images reported in Figure 3a show the spherical morphology of the silica‐based microparticles with high monodispersity and a narrow size distribution. The particle diameter is consistently around 974 ± 4 nm, with minimal variation across the field of view. Their smooth and well‐defined surfaces confirm successful synthesis and indicate good structural integrity without evident defects. Airyscan confocal super‐resolution measurements were performed to localize the reference and sensing dyes and to evaluate their distribution in the assembled sensors. The co‐localization of FITC and RBITC on an external ring around the silica core particles was confirmed (Figure 3b). Hydrodynamic diameter of the pH sensors obtained by profile analyses on FITC/RBITC maximum intensities was of 1018 ± 3 nm. This distance was in accordance with diameters calculated from SEM analyses (Figure 3a). Next, the response of the sensors to pH variations was evaluated by means of confocal laser scanning microscopy (CLSM) (Figure 3c) and by fluorimeter analyses (Figure 3d) at day 0 and 7 in DMEM F‐12 pH‐adjusted medium across physiological pH values (range 4.0–7.0). Cycles of pH switches were performed as well to assess sensors reversibility at day 0 and day 7 (Figure 3e). All calibrations revealed a direct quenching of FITC fluorescence upon increasing proton concentration in cell culture media and consistent reversibility after 7 days of aging. Then, we applied a lab‐made high‐voltage microdroplet encapsulation system for embedding the pH sensors and patient‐derived PDAC cells into alginate‐based tumorspheres. SEM micrographs (Figure 3f) showed the morphology of PDAC patient‐derived cells and pH sensors entrapped in a regular porous polymer network. The morphology of the obtained sensing‐integrated tumorspheres were also investigated by CLSM (Figure 3g). They appeared smooth and spherical in shape with the pH sensors and cells dispersed within them. Finally, the whole system was calibrated by means of CLSM. Micrographs of a representative sensing‐integrated tumorsphere exposed to different pH‐adjusted cell media (range 4.0‐7.0) are shown in Figure 3h. The calibration curve reporting the intensity ratio (I_FITC_/I_RBITC_) of FITC (pH‐indicator probe) and RBITC (reference dye) as a function of pH (Figure 3i), indicates a linear correlation between fluorescence intensity and protons concentration, and was used to extrapolate the local extracellular pH values associated with individual cells recorded during the subsequent time‐lapse experiments. Calibrations performed over a wider pH range (3.5–8.5) revealed a sensitivity range of 2.9–8.5 and a pKa of 5.7 for the fluorescein conjugated to the silica microsensor. Based on the imaging settings used during monitoring, the spatial pH resolution was 0.21 µm per pixel (Figure S4).

**FIGURE 3:**
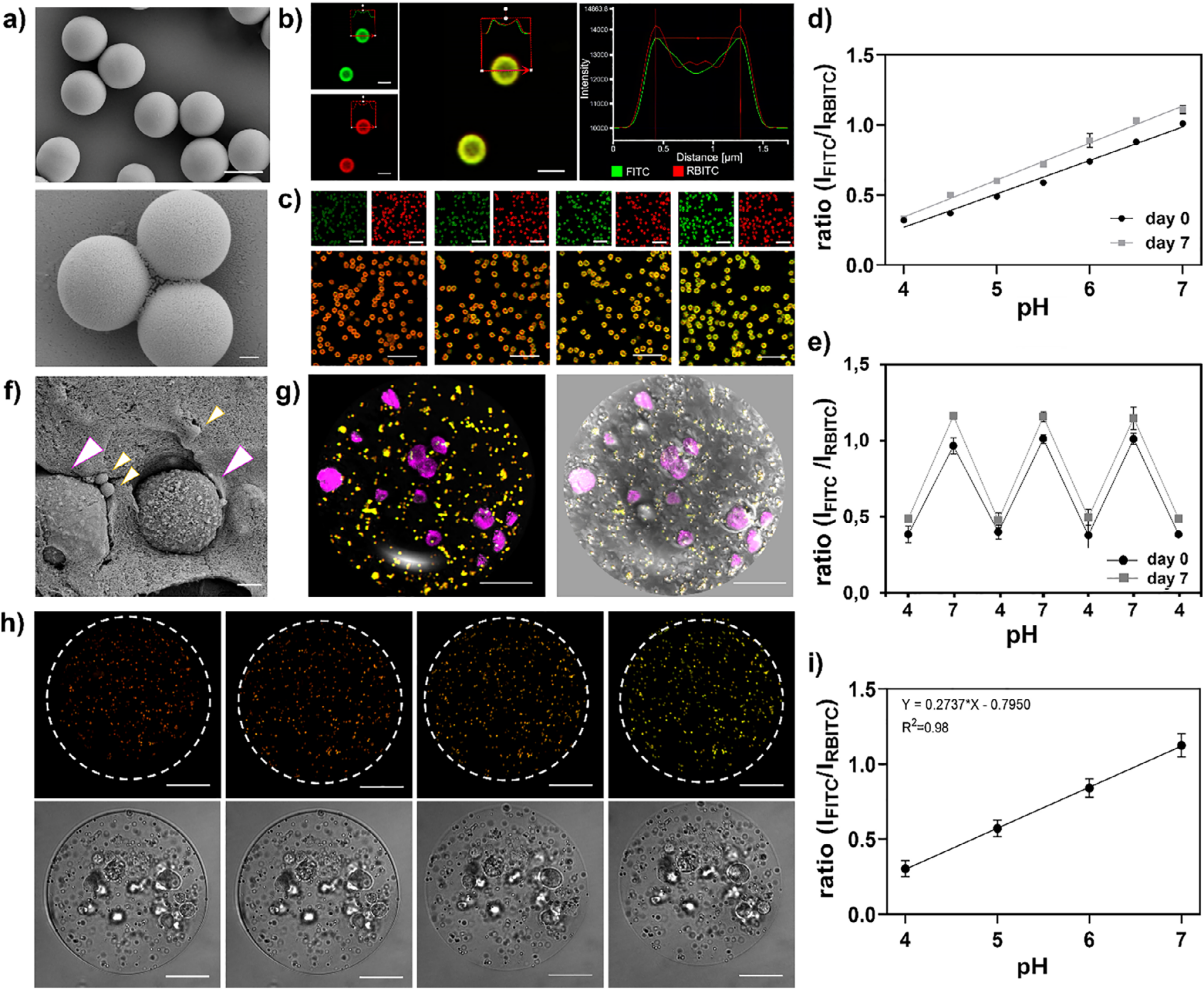
Physico‐chemical characterizations of optical ratiometric pH sensors: (a) Representative SEM images of pH sensors at low and high magnifications. Scale bars: 1 µm and 200 nm, respectively and (b) Airyscan super‐resolution imaging of pH sensor morphology showing green (FITC), red (RBITC), overlay channels, and their corresponding fluorescence intensities profiles (green channel λ_EX FITC_ = 488 nm, λ_EM FITC_ = 500–700 nm; red channel λ_EX RBITC_ = 543 nm, λ_EM RBITC_ = 556–700 nm). Scale bars: 1 µm. (c) Representative CLSM micrographs of pH sensor calibration in the pH range 4.0–7.0 (pH resolution 1.0) showing green (FITC), red (RBITC) and overlay channels (green channel λ_EX FITC_ = 488 nm, λ_EM FITC_ = 500–700 nm; red channel λ_EX RBITC_ = 543 nm, λ_EM RBITC_ = 556–700 nm). Scale bars: 5 µm. (d) Calibration curve at day 0 and day 7 of pH sensors exposed to DMEM F‐12 pH‐adjusted medium (range pH 4.0–7.0) obtained by microplate reader (λ_EX FITC_ = 472 ± 16 nm, λ_EM FITC_ = 500–700 nm; λ_EX RBITC_ = 550 ± 8 nm, λ_EM RBITC_ = 570–700 nm, ratio calculated as λ_EM MAX FITC_ = (525 nm)/λ_EM MAX RBITC_ = (570 nm)). Values expressed as mean ± SEM. (e) Spectrofluorimetric reversibility of pH sensor based on three pH switching cycles (pH 4.0 to 7.0) at day 0 and day 7 (λ_EX FITC_ = 472 ± 16 nm, λ_EM FITC_ = 500–700 nm; λ_EX RBITC_ = 550 ± 8 nm, λ_EM RBITC_ = 570 nm ± 10 and 700 ± 10; ratio calculated as λ_EM MAX FITC_ = (525 nm)/λ_EM MAX RBITC_ = (570 nm)). Values expressed as mean ± SEM. (f) SEM image of an internal section of the alginate‐based tumorsphere embedding pH sensors (yellow arrow), patient‐derived PDAC cells (magenta arrow). Scale bars, 2 µm, respectively. (g) Representative CLSM 3D reconstruction of sensing‐integrated tumorsphere embedding pH sensors (yellow, false color) and PDAC patient‐derived cells (magenta, false color, representative of Patient 1); (green channel λ_EX FITC_ = 488 nm, λ_EM FITC_ = 500–700 nm; red channel λ_EX RBITC_ = 543 nm, λ_EM RBITC_ = 556–700 nm; magenta channel λ_EX deep red_ = 639 nm, λ_EM deep red_ = 640–700 nm). Scale bars: 50 µm. (h) Representative CLSM images of sensing‐integrated tumorsphere embedding pH sensors and PDAC patient‐derived cells (representative of Patient 1) exposed to different pH‐adjusted cell media (pHs 4.0, 5.0, 6.0, 7.0). FITC (green channel)/RBITC (red channel) overlays and PDAC patient‐derived cells in bright field (grey channel) are reported. Maximum z‐projections were extrapolated from 28 z‐stacks for each pH. Z‐stack step size = 2.55 µm. Scale bars, 50 µm. (i) Calibration curve of pH sensors reporting the fluorescence intensity ratio of FITC (green channel) and RBITC (red channel) as function of pH. Data are means ± SEM of three independent experiments (*n* = 3).

### 2.4 Extracellular pH Monitoring in Patient‐Derived Tumorspheres Reveals Patient‐ and Drug‐Specific Local Heterogeneity

The acidic microenvironment is a hallmark of PDAC and arises from a metabolic switch toward aerobic glycolysis (the so‐called “Warburg effect”) that favors rapid energy production and tumor growth [48]. This shift leads to the excessive lactate and proton release, even under normoxic conditions. As a result, the extracellular microenvironment becomes highly acidic, supporting the elevated energy demands of quickly proliferating cancer cells [49]. This tumor acidosis is linked with other cancer hallmarks including invasion, metastasis, and chemoresistance against the standard therapy of PDAC [50]. Furthermore, the metabolic alterations of glucose, amino acids, lipids, and other metabolites, are key factors contributing to the disheartening outcomes of clinical trials targeting PDAC [51]. Importantly, the patients’ prognosis and the successful response to chemotherapy are impacted by a distinct metabolic heterogeneity existing in PDAC, such as different pH gradients established at single‐cell level within the tumor tissue [52]. Non‐invasive measurements of these local pH variations, particularly during drug exposure, are therefore essential for understanding treatment outcomes and stratifying patients. To this end, we developed alginate‐based tumorspheres, embedding patient‐derived PDAC cells from five independent specimens together with ratiometric optical pH sensors to map extracellular pH dynamics associated with individual cells during chemotherapy (Figure 4). Each tumorsphere was treated with pharmacologically relevant concentrations of FOLFIRINOX, gemcitabine, or paclitaxel, determined from the IC_50_ values of each drug in matched patient‐derived cells (Figure S5a–f). CLSM time‐lapse experiment was then performed over 12 h in a temperature‐ and CO_2_‐controlled chamber to capture spatio‐temporal local extracellular pH fluctuations during treatment. The schematic illustration of the experimental set‐up is reported in Figure 4a, showing representative CLSM time‐lapse images recorded at 0 h (T0) and 12 h (T11) of a pH sensing tumorspheres (left panel). In the right panel, representative 3D scatter plots of pH sensor signals and PDAC patient‐derived cells extrapolated from the CLSM measurements of the whole tumorspheres (left) and as local extracellular pH readouts associated with individual cells (right) were reported at 0 h (T0) and 12 h (T11). An automated computational approach was applied for analyzing the time‐lapse experiments, and the local extracellular pH fluctuations associated with individual cells recorded during exposure to FOLFIRINOX, gemcitabine, and paclitaxel were tracked. To confirm that local pH variations originated exclusively from cellular metabolism rather than drug administration, control experiments were performed by comparing the pH of DMEM F‐12 medium in the absence and presence of the highest drug concentrations employed during the monitoring of patient‐derived tumorspheres. Fluorimetric analyses revealed no measurable acidification following drug addition, with pH values remaining comparable to those of the untreated DMEM F‐12 control (Figure S6).

**FIGURE 4:**
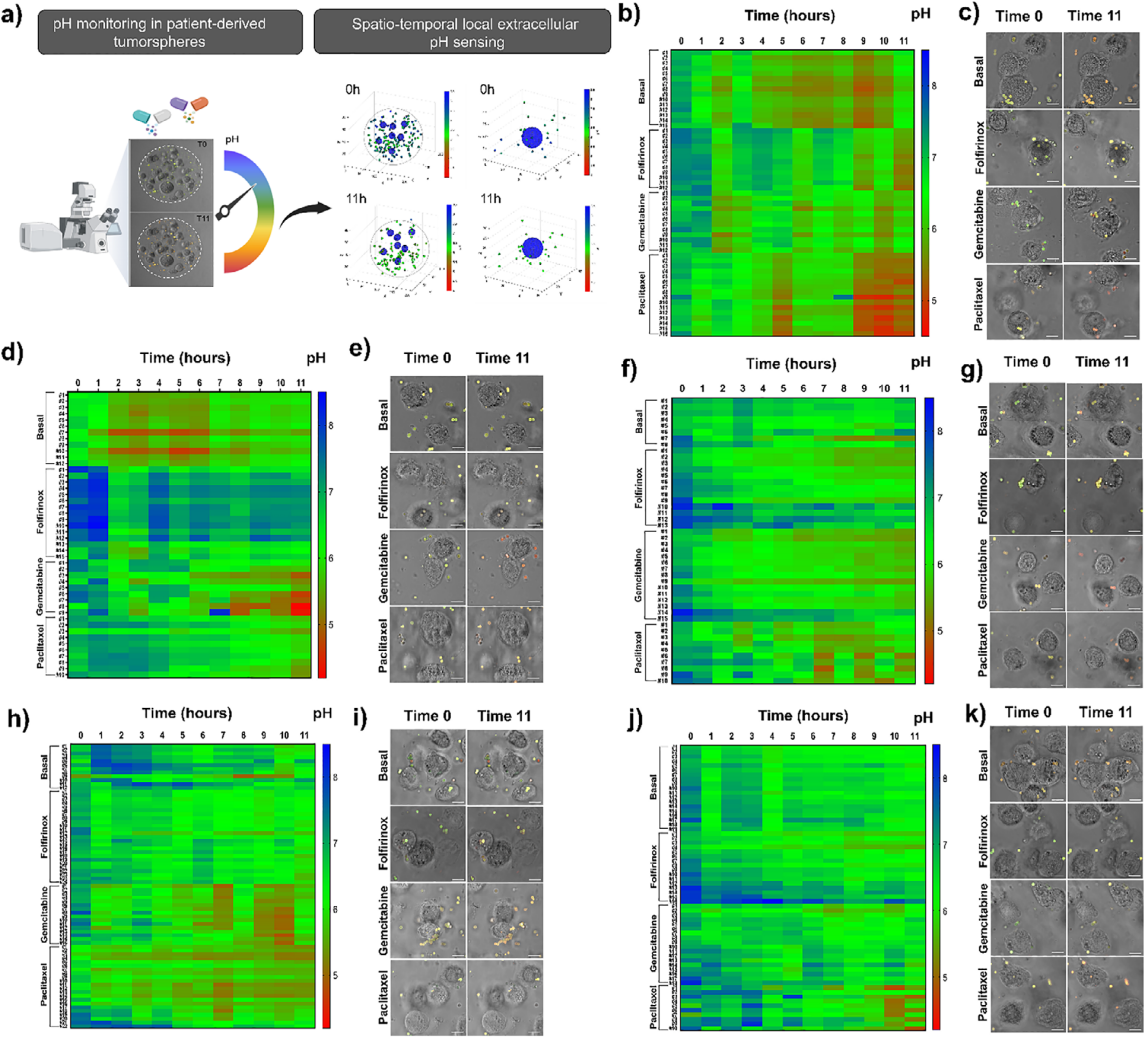
Extracellular pH monitoring over time and space in patient‐derived tumorspheres during drug testing. (a) Schematic illustration of the experimental setup (left panel) and representative CLSM time‐lapse images of an alginate‐based tumorsphere embedding pH sensors and patient‐derived PDAC cells (right). Images were acquired at baseline (T0) and after 12 h (T11) during exposure to chemotherapeutic agents. Overlay of FITC (pH‐sensitive, green), RBITC (reference, red), and bright field (gray) were merged to visualize sensors and cells. Representative 3D scatter plots (right panel) of pH sensor signals and PDAC patient ‐derived cells extrapolated from the CLSM measurements recorded at T0 and T11. Extracellular pH variations were monitored and quantified over time and space in the whole tumorsphere (left) and as cell‐associated local extracellular pH values (right), calculated from 3D‐reconstructed pH sensors located in proximity to individual cells through the image‐analysis pipeline. Objects are not drawn on scale. (b,d,f,h,j) Heatmaps of extracellular pH variations over time associated with individual cells in sensing‐integrated tumorspheres during drugs testing for patients 1–5. Each rectangle represents the pH of a single cell, color‐coded from blue (alkaline, pH 7.5–8) to green (neutral, pH 5.5–7.5) to red (acidic, pH 4–5.5). (c,e,g,i,k) Representative CLSM z‐projection images (maximum intensity from 27 z‐stacks, 1 µm step size) of cells surrounded by fluorescent pH sensors at T0 and T11, illustrating patient‐ and drug‐specific extracellular acidification patterns. Scale bars, 8 µm. Data represent means ± SEM from triplicate experiments (*n* = 3).

In Figure 4b–j, the colorimetric heatmaps of local extracellular pH variations over time associated with individual cells were reported for five PDAC patients. These data derived from CLSM time‐lapse measurements of tumorspheres embedding fluorescent pH sensors and patient‐derived cells dissociated from each PDAC tissue, in both untreated (Basal) and treated with FOLFIRINOX, gemcitabine, and paclitaxel. Each rectangle indicates the pH value associated with one cell, and the color varies from blue (basic pH 7.5‐8) to green (5.5‐7.5), to red (acidic pH 5.5‐4) according to the pH fluctuations. A strong heterogeneity in local extracellular pH values associated with individual cells was observed for each patient. In fact, in Figure 4c,e,g,i,k reporting CLSM images of individual cells surrounded by fluorescent pH sensors, it was possible to appreciate that in all PDAC patients distinct local extracellular pH fluctuations were detected around different cells, as indicated by the change in fluorescence of the pH sensors, in both basal conditions and during chemotherapy treatment. Interestingly, the extracellular acidification was patient‐specific and varied depending on the drug administered. It was evident that patients 1, 3, and 5 showed an acidic profile after treatment with paclitaxel, reaching pH values between 5.5 and 4.5, whereas patient 2 displayed a stronger acidification after treatment with gemcitabine. Moreover, patient 4 responded with an acidic pH after treatment with both paclitaxel or gemcitabine. It is important to note that this strongly acidic extracellular pH recorded in standard culture media over short times corresponds to highly localized measurements associated with a small subset of cells within the tumorpheres after treatment with chemotherapeutic agents. Moreover, the recorded pH values were not representative of the bulk extracellular environment. This phenomenon may arise from transient metabolic stress, increased glycolytic activity, or impaired proton diffusion within the alginate matrix after chemotherapeutic treatments. Notably, the heterogenous pH values associated with individual cells were less evident in bulk measurements based on average values for entire tumorspheres (Figure S7). These results highlight the functional significance of spatial pH mapping in our platform, as it enables the identification of local extracellular acidification patterns at the level of individual cells within the 3D tumorsphere. Because pH values were calculated from sensors located within a defined distance from each cell, this readout reflects a local extracellular microenvironment that may also include contributions from neighboring cells, particularly in dense 3D regions. Importantly, this approach revealed marked cell‐to‐cell heterogeneity during drug exposure that was substantially attenuated when pH values were averaged over the whole tumorsphere (Figure S7). Taken together, these findings demonstrate that sensing‐integrated tumorspheres enable mapping of local extracellular acidification associated with individual cells, revealing patient‐ and drug‐specific metabolic responses that are less evident in bulk measurements and a functional link between pH metabolic heterogeneity and therapeutic outcome.

### 2.5 Chemotherapeutic Agents Differentially Induce Apoptosis, Lactate Secretion, and Reduce Proliferation in Patient‐Derived Tumorspheres

Chemotherapeutic treatments profoundly influence tumor cell metabolism and survival, often leading to changes in extracellular acidification that reflect metabolic stress and cell death [53]. In particular, chemotherapeutic agents that cause DNA damage can trigger cell death mechanisms, such as apoptosis [54]. It is known that FOLFIRINOX, gemcitabine and paclitaxel activate intrinsic and extrinsic apoptotic pathways [55, 56, 57]. These processes are accompanied by plasma membrane disruption, loss of mitochondrial integrity, and release of acidic intracellular contents, which further contribute to tumor microenvironment acidification [58]. Besides chemotherapy‐induced cellular damage, lactate production and secretion by metabolism‐reprogrammed tumor cells also cause a significant extracellular acidification within the tumor microenvironment providing a direct correlation with the cellular metabolic state [59, 60].

The above reported pH‐sensing analyses revealed that within 12 h, the local extracellular acidification patterns at the level of individual cells in all PDAC patients was different in both basal condition and during chemotherapy treatment. Moreover, the extracellular acidification was patient‐specific and varied depending on the drug administered. To determine whether these pH fluctuations were associated with cell death events, we evaluated phosphatidylserine exposure, a well‐established early marker of apoptosis, using Annexin V–FITC and propidium iodide (PI) staining. Tumorspheres from all five patients were analyzed under basal conditions and following 12 h of treatment with FOLFIRINOX, gemcitabine, or paclitaxel (Figure 5a,c,e,g,i). Importantly, these flow cytometric analyses were performed as an independent functional validation of the pH‐sensing platform rather than as a spatial readout. As tumorsphere dissociation is an intrinsic requirement for flow cytometry, spatial information is inevitably lost, and the direct correlation between local extracellular pH and spatially resolved biological readouts is not feasible. Representative flow cytometry dot plots illustrate the proportion of viable cells (Annexin V^−^/PI^−^), early apoptotic cells (Annexin V^+^/PI^−^), late apoptotic cells (Annexin V^+^/PI^+^), and necrotic cells (Annexin V^−^/PI^+^) for each treatment. All three chemotherapeutic agents significantly increased the frequency of apoptotic and necrotic cells compared to basal conditions, where spontaneous cell death was minimal. However, the magnitude and pattern of these responses were highly patient‐specific.

**FIGURE 5:**
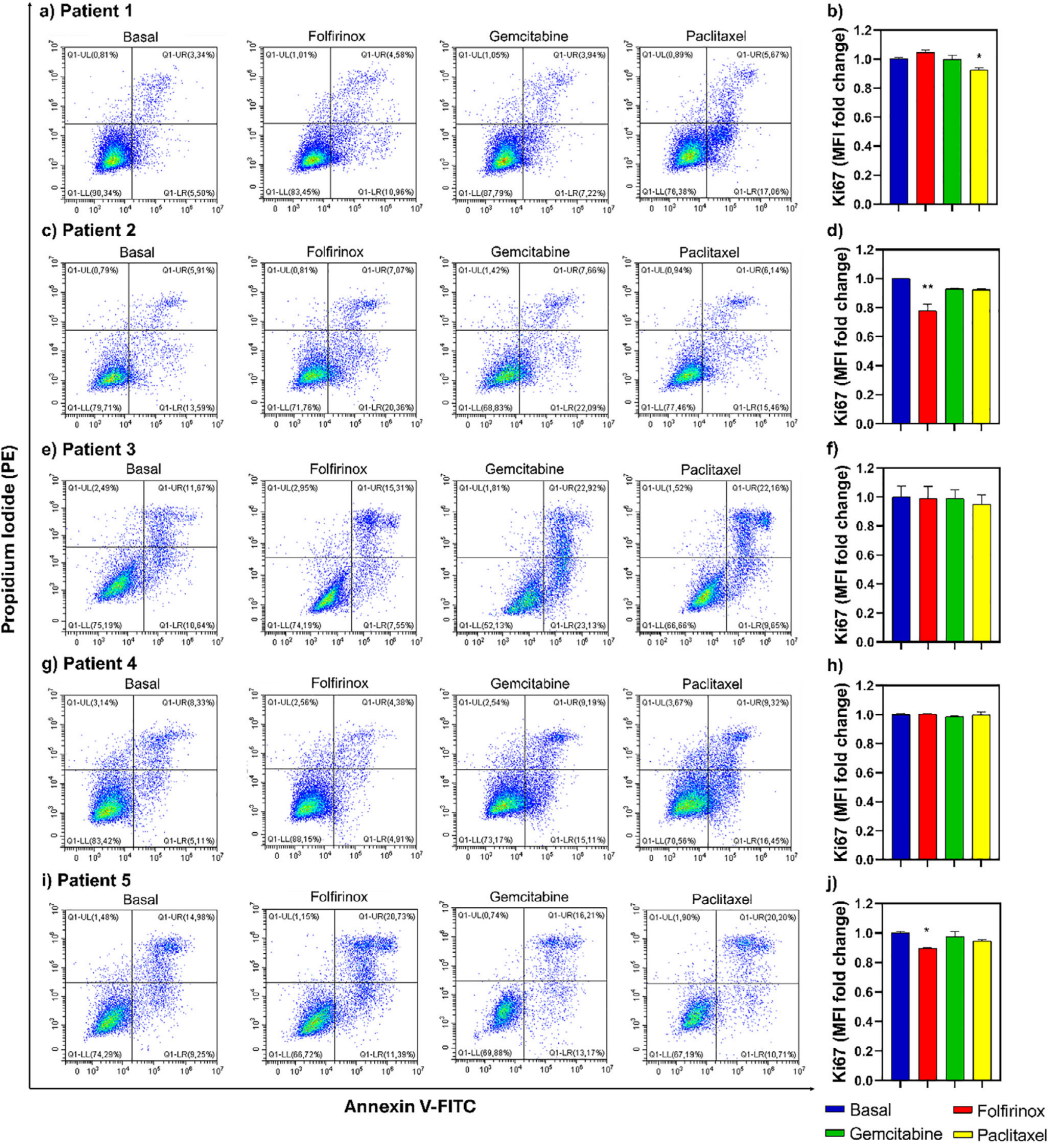
Apoptotic cell death and proliferation in patient‐derived tumorspheres after chemotherapy. (a,c,e,g,i) Representative flow cytometry dot plots analyzed of patient‐derived tumorspheres treated with folfirinox, gemcitabine and paclitaxel for 12 h, stained with Annexin V‐FITC (x‐axis) and PI (y‐axis). Q1‐LR, Q1‐UR and Q1‐UL quadrants indicate early apoptosis (Annexin V^+^/PI^−^), late apoptosis (Annexin V^+^/PI^+^), necrosis (Annexin V^−^/PI^+^), and viable cells (Annexin V^−^/PI^−^). (b,d,f,h,j) Ki‑67 expression levels (median fluorescence intensity, MFI) in untreated (Basal) and treated tumorspheres for each patient, measured after 12 h of exposure. Data are mean ± SEM from triplicate experiments (*n* = 3). The statistical analysis was performed using one‐way analysis of variance (ANOVA): ^*^ *p* < 0.01, ^**^ *p* < 0.001, treated *vs* basal conditions.

For example, paclitaxel strongly induced apoptosis in tumorspheres from patients 1 (Figure 5a) and 4 (Figure 5g), as evidenced by a large increase in Annexin V^+^ populations. In contrast, FOLFIRINOX was the main trigger of apoptosis in patients 2 (Figure 5c) and 5 (Figure 5i), with elevated levels of both early and late apoptotic cells. For patient 3 (Figure 5e) gemcitabine was the most potent inducer of cell death. These drug‐specific apoptotic profiles correlated closely with the extracellular acidification patterns previously observed by pH‐sensing analysis. Patients whose tumorspheres exhibited more pronounced acidification during treatment also displayed higher levels of apoptosis, suggesting that the acidification phenotype reflects, at least in part, cell death–associated metabolic collapse. To further assess the anti‐proliferative effects of the three chemotherapeutic agents, we quantified the expression of Ki‑67, a nuclear protein expressed in all active phases of the cell cycle but absent in quiescent (G0) cells. Ki‑67 median fluorescence intensity (MFI) was measured by flow cytometry after 12 h of drug exposure (Figure 5b,d,f,h,j). In patients 1 (Figure 5b), 2 (Figure 5d), and 5 (Figure 5j), the induction of apoptosis was accompanied by a substantial reduction in Ki‑67 expression, consistent with a strong inhibition of proliferation. By contrast, Ki‑67 levels remained largely unchanged in patients 3 (Figure 5f) and 4 (Figure 5h), aligning with their lower levels of apoptosis in response to the tested drugs. To further support these data and correlate them with a more direct measure of the cellular metabolic state, the extracellular lactate quantification in patient‐derived tumorspheres under basal conditions and following drug treatments were performed at 1, 6, and 12 h (Figure S8). These data demonstrated that lactate secretion profiles were highly patient‐ and drug‐specific and showed a correlation with the extracellular pH dynamics measured by the sensing‐integrated platform reported in Figure 4. In particular, drug treatments inducing stronger extracellular acidification were generally associated with increased lactate accumulation, supporting the interpretation that pH fluctuations are not exclusively linked to apoptotic cell death but also reflect dynamic metabolic reprogramming and glycolytic heterogeneity within patient‐derived PDAC tumorspheres. In general, this correlation was particularly evident for patients 2, 4 and 5. Specifically, in the sensing‐integrated tumorspheres derived from patients 2 and 4, gemcitabine treatment for 12 h induced greater local acidification, which also resulted in higher lactate secretion compared to basal conditions, whereas for patient 5, this correlation was observed following paclitaxel treatment. Importantly, the lactate measurements also revealed that acidification and lactate production were not always perfectly correlated across patients 1 and 3, suggesting that extracellular pH dynamics likely result from the combined contribution of glycolytic metabolism, proton extrusion mechanisms, and treatment‐induced cell death. Taken together, these findings demonstrate that chemotherapeutic agents induce distinct, patient‐specific apoptotic and proliferative responses, as well as lactate secretion profiles in PDAC tumorspheres. The correlation between extracellular acidification dynamics, a more direct measure of glycolytic activity through lactate quantification, and apoptosis suggests that real‐time pH monitoring can serve as a functional readout of drug‐induced cytotoxicity and better supporting the interpretation of inter‐patients metabolic heterogeneity.

Moreover, the integrated approach, combining sensing‐enabled tumorspheres and multiparametric flow cytometry, provides a powerful framework for stratifying patients based on their metabolic and apoptotic responses to chemotherapy.

### 2.6 Correlation between In Vitro Drug Response of Patient‐Derived Sensing‐Integrated Tumorspheres and Clinical Outcomes

To evaluate the predictive value of sensing‐integrated patient‐derived tumorspheres, we correlated their in vitro drug response profiles with the clinical outcomes of the corresponding PDAC patients (Table 1). Each tumorsphere was classified as *resistant*, *moderately sensitive*, or *sensitive* to FOLFIRINOX, gemcitabine, or paclitaxel based on the degree of cell death measured after 12 h of treatment (Figure 5a,c,e,g,i) and the associated extracellular acidification patterns (Figure 4). In line with previous studies reporting moderate extracellular acidity (pH ∼6.6) in PDAC, with localized regions of severe acidity (pH < 6.5) particularly in hypoxic tumor cores or following chemotherapy, [21, 50, 61] we stratified the metabolic response of tumorspheres into mild (pH 7.0–6.0), moderate (pH 6.0–5.0), or severe acidification (pH 5.0–4.0). This classification was derived from local extracellular pH measurements associated with individual cells at 12 h of treatment for each patient. Patient 1 could not be evaluated clinically due to an unfavorable post‐surgical outcome unrelated to cancer; however, in vitro testing revealed higher sensitivity to paclitaxel compared to FOLFIRINOX, and resistance to gemcitabine, correlating with severe and moderate extracellular acidification, respectively. Patient 2 showed resistance to FOLFIRINOX in vitro but remained disease‐free 22 months after surgery following 12 cycles of this regimen, representing the only case where in vitro predictions did not fully match clinical outcome. Patients 3 and 4 were predicted to be sensitive to gemcitabine, which correlated with severe‐to‐moderate extracellular acidification linked to extensive drug‐induced cell death. These predictions were fully consistent with the clinical decision to administer gemcitabine (12 cycles for patient 3 and 6 cycles for patient 4); both patients remained disease‐free 20 and 16 months after surgery, respectively. Finally, patient 5 was classified as resistant to all tested treatments based on low cell death and minimal extracellular acidification in tumorspheres. Clinically, this patient received 6 cycles of gemcitabine + paclitaxel followed by 6 cycles of FOLFIRINOX but developed liver recurrence 6 months after surgery, in line with the in vitro predictions. To directly assess whether local pH measurements provide additional predictive information over conventional bulk measurements, we performed the correlation analysis using the average extracellular pH of the whole tumorspheres. Although the limited cohort size (*n* = 5) precluded formal statistical analysis, a descriptive comparison showed that, in most treatment conditions, bulk pH measurements reproduced the overall acidification trend but attenuated the magnitude of the response by averaging local pH gradients. In contrast, in most cases, cell‐associated pH measurements identified localized regions of severe acidification that were not evident from bulk values and more closely reflected the drug‐response profiles and clinical outcomes. Furthermore, establishing a predefined correspondence between acidification severity and drug‐response category (mild acidification was classified as resistant, moderate acidification as moderately sensitive, and severe or severe‐to‐moderate acidification as sensitive), cell‐associated local pH measurements agreed with the tumorsphere drug‐response classification in 10 of 15 patient–drug combinations (66.7%), compared with 7 of 15 combinations (46.7%) for bulk pH measurements. This descriptive analysis supports the additional predictive information provided by spatially resolved pH measurements. Comparisons with clinical outcomes were interpreted at the patient and treatment‐regimen level, because combination and sequential therapies precluded the unambiguous attribution of clinical outcome to individual drugs. Overall, this analysis demonstrates that the experimental workflow combining cell death quantification and cell‐associated extracellular pH monitoring in patient‐derived tumorspheres can predict clinical outcomes with a high degree of accuracy. Although our cohort size was limited (n  =  5), these findings support the potential of sensing‐integrated tumorspheres as a clinically relevant platform for personalized drug screening. Larger patient cohorts will be essential to validate the predictive power of this approach and assess its integration into precision oncology workflows.

**TABLE 1:** Correlation of sensing‐integrated tumorspheres predictions with clinical outcomes in PDAC patients. Tumorspheres were generated from surgical specimens of five PDAC patients and treated with FOLFIRINOX, gemcitabine, or paclitaxel at patient‐specific IC_50_ concentrations. Clinical data include adjuvant chemotherapy regimens (number of chemotherapy cycles and drug combinations) and oncological outcomes (disease‐free survival or recurrence). Tumorspheres predictions are classified as *resistant*, *moderately sensitive*, or *sensitive* based on each patient's response to the drugs, considering the cell death measured after 12 h of treatment. The acidification features are derived from local or bulk extracellular pH values registered at 12 h of treatment for each patient indicated as mild (pH 7‐6), moderate (pH 6‐5) or severe (pH 5‐4); (*) denotes the cell‐associated local pH feature and (#) denotes the average bulk pH.

Patient ID			Folfirinox			Gemcitabine			Paclitaxel		
	Adjuvant chemotherapy	Oncological outcome	Tumorspheres prediction	Adjuvant	Acidification feature (*local pH; #bulk pH)	Tumorspheres prediction	Adjuvant	Acidification feature (*local pH; #bulk pH)	Tumorspheres prediction	Adjuvant	Acidification feature (*local pH; #bulk pH)
#1	—	Dead at 2 mo. post surgery for non oncological reason	Moderately sensitive	—	*#Moderate	Resistant	—	*#Moderate	Sensitive	—	*Severe #Moderate
#2	12 cycles	Disease free at 22 mo.	Resistant	Yes	*Mild #Moderate	Sensitive	—	*#Severe	Resistant	—	*#Moderate
#3	12 cycles	Disease free at 20 mo.	Resistant	—	*Mild #Moderate	Sensitive	Yes	*Severe‐moderate #Moderate	Resistant	—	*Severe‐moderate #Moderate
#4	6 cycles	Disease free at 16 mo.	Resistant	—	*Mild #Moderate	Moderately sensitive	Yes	*Severe‐moderate #Moderate	Moderately sensitive	—	*#Moderate
#5	6 cycles + 6 cycles	Liver recurrence at 6 mo.	Resistant	Yes	*#Mild	Resistant	Yes	*#Mild	Resistant	Yes	*Severe‐moderate #Mild

## 3 Conclusions

Over the last years, the primary strategies in precision oncology to drive patient treatment have been genomic profiling and biomarker identification [62]. While this approach has led to breakthroughs in subsets of malignancies, recent data indicate that less than 10% of cancer patients derive a durable clinical benefit from genomics‐driven targeted therapy [63]. This limited success stems from multiple factors: the absence of clinically relevant mutations in many tumors, the functional redundancy of oncogenic pathways, and the emergence of adaptive resistance mechanisms even in patients harboring targetable alterations [64, 65]. In pancreatic ductal adenocarcinoma (PDAC), the situation is particularly challenging. This disease is characterized by a dense desmoplastic stroma, extensive intra‐ and inter‐tumor heterogeneity, and profound metabolic plasticity, all of which undermine the predictive value of genomics alone [66]. Recent single‐cell studies have highlighted how dynamic cell–cell and cell–matrix interactions within the tumor microenvironment (TME) drive phenotypic plasticity, therapeutic evasion, and chemoresistance [66]. As a result, despite rapid advances in validated drug targets and novel agents [67], a robust and reproducible functional precision medicine approach for PDAC is still lacking. There is increasing recognition that patient‐derived tumor models could bridge this gap by complementing molecular profiling with phenotypic drug testing. Several organoid and xenograft platforms have shown promise in recapitulating tumor architecture and predicting clinical response [31, 34, 35]. However, these systems rarely integrate real‐time functional readouts of the TME, particularly metabolic dynamics such as extracellular acidification, which are key modulators of therapy efficacy [68]. Identifying non‐responsive tumors in vitro would not only spare patients from ineffective treatment but also enable the testing of novel drug combinations within clinically actionable timelines. Here, we present a sensing‐integrated, patient‐derived tumorsphere platform that allows simultaneous drug screening and high‐resolution mapping in local extracellular regions associated with individual cells. By embedding dissociated primary PDAC cells from five patients within an alginate‐based 3D matrix doped with optical ratiometric pH sensors, we developed a preclinical model capable of real‐time, non‐invasive monitoring of extracellular acidification during chemotherapy. Our findings reveal a pronounced patient‐specific heterogeneity in extracellular acidification patterns, with distinct cell‐associated local pH that correlated with apoptotic responses and, in most cases, with clinical outcomes. Such heterogeneity was masked by bulk pH measurements, underscoring the importance of local extracellular pH analysis for capturing subtle but clinically relevant metabolic phenotypes. Notably, the extracellular pH acidification was measured at the collective multicellular level within the whole PDAC tumorspheres, in which epithelial tumor cells, stromal/mesenchymal and immune populations coexist and dynamically interact within a shared microenvironment. In this regard, preserving multicellular interactions and collective metabolic behavior represents a key advantage of the sensing‐integrated patient‐derived tumorsphere, since PDAC therapeutic resistance is increasingly recognized as an emergent consequence of the dynamic cell‐cell interactions. However, another important aspect that could further strengthen the relevance of the platform in future studies is the correlation of the local pH variations with specific cell populations preferentially associated with more or less acidic regions.

This work builds upon recent evidence that the acidic TME not only promotes tumor progression but also impairs the delivery and efficacy of chemotherapeutic agents. This methodology could offer a powerful avenue for functional precision medicine in PDAC by predicting therapeutic responses in a timeframe shorter than traditional clinical assessments. Future work should aim to validate this approach in larger patient cohorts and to integrate it with emerging omics data, multiplex imaging, and computational modeling to generate multi‐parametric predictors of treatment outcome.

Additionally, adapting this sensing‐integrated platform for high‐throughput drug screening could accelerate the identification of novel therapeutic combinations that overcome the formidable resistance mechanisms characteristic of PDAC [69].

## 4 Methods

### 4.1 Reagents and Resources

Chemicals: Rhodamine B isothiocyanate (RBITC), Fluorescein 5(6)‐isothiocyanate (FITC), Alginic acid sodium salt from brown algae, Calcium chloride dihydrate, Irinotecan hydrochloride (IRI), Oxaliplatin (OXA), and 5‐Fluorouracil (5‐FU) were all purchased from Sigma‐Aldrich, Darmstadt, Germany. Gemcitabine HCl was purchased from Selleckchem, Paclitaxel from Thermofisher Scientific. Tetraethyl orthosilicate (TEOS) and (3‐Aminopropyl) triethoxysilane (APTES) were purchased from Aldrich chemistry. Anhydrous ethanol from VWR, ethanol from Honeywell, Potassium Chloride (KCl) from Sigma life science. Dulbecco's Modified Eagle's Medium/Nutrient Mixture F‐12 Ham, (DMEM F‐12, Cat#SD6421), FBS (Cat#F7524), L‐Glutamine solution (Cat#G7513), Penicillin‐Streptomycin (PEN/STREP, Cat#P0781), Amphotericin B (Cat#A2942), Recombinant Human Fibroblast Growth Factor‐basic (FGFb, Cat#PHG0024) were purchased from Sigma‐Aldrich, Darmstadt, Germany.

Antibodies: FITC anti‐human CD324 (E‐cadherin, Cat#324104), APC anti‐human CD326 (EpCAM, Cat#369810), PE anti‐human CD325 (N‐Cadherin, Cat#350805), PE anti‐human Ki‐67 (Cat#350504), Alexa Fluor‐488 anti‐Vimentin Antibody (Cat#699306), Alexa Fluor‐647 anti‐Cytokeratin (pan reactive) Antibody (Cat#628604), APC Fire‐750 anti‐human CD45 Antibody (Cat#304061) were all purchased from BioLegend, United Kingdom. PE anti‐human CD14 (Cat#130‐110‐519) was purchased from Miltenyi Biotec, Bergisch Gladbach, Germany, whereas Alexa Fluor‐594 Anti‐hFAP Antibody (Cat#FAB3715T) was purchased from R&D Systems, Bio‐Techne, Italy.Commercial kits: Tumor Dissociation Kit, Human (Cat#130‐095‐929), MACS Tissue storage solution (Cat#130‐100‐008) were purchased from Miltenyi Biotec, Bergisch Gladbach, Germany. Human patient material is provided by Ospedale “Vito Fazzi”, Lecce.

### 4.2 Collection of Fresh Human PDAC Tissues

Human PDAC tissues were obtained from male or female patients using the tissue collection protocol (n. 32577/2023) that is approved by the ASL Lecce Ethical Committee (DGR n.601/2022, DGR 683/2022 project number 13). PDAC patients enrolled in this study were from “Ospedale Vito Fazzi” (Lecce, Italy), and all procedures were conducted in accordance with the ethical principles for medical research involving human subjects. All patients provided their informed consent to the referring physician or to other clinicians involved in the study. According to the Pathology Unit of the “Vito Fazzi” Hospital, all surgical samples were confirmed to be PDAC at different stages before processing. Clinicopathological data, including tumor grade, stage, primary site, metastatic status, and tumor cellularity, were collected for all PDAC patients included in the study. Tumor cellularity and stromal content were assessed on hematoxylin and eosin (H&E)‐stained sections at 20X magnification across 10 high‐power fields (HPFs).

### 4.3 Human PDAC Tissues Processing and Cell Cultures

During surgery a small piece (∼0.5–1 cm^3^, ∼0.5–1 g) of pancreatic tumor was cut from the specular region to that used for the histological examination. Each tissue sample was kept in the MACS Tissue Storage Solution (Miltenyi Biotec, Germany) at 4°C until dissection, and then it was washed with PBS 1X and cut into small pieces by removing fat, fibrous, and necrotic areas. Minced tissue samples were then dissociated by using the human Tumor Dissociation kit (Miltenyi Biotec., Germany), following the manufacturer's protocol. Briefly, the tissue pieces were transferred into a gentleMACS C tube containing the enzyme mix, consisting of Enzyme H (200 µl), R (100 µl), and A (25 µl) dissolved in 4.7 ml of RPMI1640. Minced tissue samples were then digested through a mechanical dissociation by running the gentleMACS program h_tumor_01 on the gentleMACS Dissociator (Miltenyi Biotec., Germany) combined with three cycles of enzymatic degradation under gentle agitation for 30 min at 37°C. If large clumps were observed, an additional program for tough tumors was run. After digestion, cells were filtered through a 70 µm MACS SmartStrainer and washed with 10 ml of PBS 1X. After centrifugation, cells were seeded in a Corning 25 cm^2^ Cell Culture Flask and grown in Dulbecco's Modified Eagle's Medium/Nutrient Mixture F‐12 Ham, (DMEM F‐12) supplemented with 10% FBS, 2 mM L‐Glutamine solution, 100 U/mL Penicillin‐Streptomycin (PEN/STREP), 1% Amphotericin B, 1µg/ml of Recombinant Human Fibroblast Growth Factor‐basic (hFGFb), 1µg/ml of Recombinant Human Epidermal Growth Factor (hEGF). Medium was changed every 3 days.

### 4.4 Patient‐Derived Cells Characterization by Flow Cytometry

Patient‐derived cell lines from 5 PDAC tissues established by day 15–20 or after thawing and three passages in culture were detached by using acutase treatment (Invitrogen, Thermo Fisher Scientific) and incubated at 37°C for 2 min. Cells were collected, washed with PBS 1X, and fixed with 2% paraformaldehyde for 15 min at 4°C. Samples were then incubated with saponin 0.5% and stained with the following antibodies at room temperature for 30 min: FITC anti‐human CD324 (E‐cadherin, Biolegend Cat#324104, 1:250), APC anti‐human CD326 (EpCAM, Biolegend Cat#369810, 1:250), PE anti‐human CD325 (N‐Cadherin Biolegend Cat#350805, 1:250), Alexa Fluor‐488 anti‐Vimentin Antibody (Biolegend Cat#699306, 1:200), Alexa Fluor‐647 anti‐Cytokeratin (pan reactive) Antibody (Biolegend Cat#628604, 1:100), Alexa Fluor‐594 Anti‐hFAP Antibody (R&D Systems Cat#FAB3715T, 1:200), APC Fire‐750 anti‐human CD45 Antibody (Biolegend Cat#304061, 1:250), PE anti‐human CD14 (Miltenyi Biotec Cat#130‐110‐519, 1:200). After incubation, cells were washed with PBS+1% FBS before the analysis with the CytoFLEX S flow cytometer (Beckman Coulter, USA). Approximately 20.000 events were acquired for each sample and analyzed using CytExpert software. Dead cells, debris, and doublets were excluded based on forward scatter and side scatter measurements. To identify the phenotypically defined cell subsets based on surface markers expression, single‐cell data was clustered, merged with singlets, and cell populations defined by the manual gating strategy, were projected onto a t‐SNE map by using FlowJo 11. Specific colors were assigned to each group based on positive cells to surface markers.

### 4.5 Synthesis and Characterization of Optical Ratiometric pH Sensors

Optical ratiometric pH sensors based on silica (SiO_2_) were obtained as previously described [27]. Briefly, the optical sensors were synthesized through a basic hydrolysis reaction by adding in the batch reactor the TEOS monomer (200 µL), the NH_4_OH catalyst (760 µL), and the KCl electrolyte (2.8 mg) to an ethanol mixture (12 mL EtOH, 1.12 mL H_2_O) to produce monodispersed silica seeds (room T, 30 min, 240 rpm). The silica seeds were further silanized to assemble optical ratiometric sensors by using APTES as linker [70] while FITC and RBITC as indicator probe and reference dye, respectively. Then, the conjugated FITC‐APTES and RBITC‐APTES were synthetized in tandem by solubilizing 0.5 mg of FITC/RBITC in 2 mL of anhydrous EtOH and adding 0,13 uL of APTES, under controlled stirring (room T, 2 h, 500 rpm). The crude products were united upon their reaction's completion, and additional 336 µL of TEOS monomer were added for silica regrowth purposes. These products were then injected into the silica seed batch reactor with a controlled flow rate of 0.05 mL/h (room T, 24 h, 240 rpm). The final product was centrifuged (2500 rpm, 5 min, room T) and washed three times, first in 50 mL of EtOH and then in distilled water. The clean pH sensors were resuspended in EtOH to obtain a 40 mg/mL stock solution and were stored in the dark at room T. The SiO_2_‐based pH sensors were then characterized as previously described [27] by evaluating their response in the range of physiological pHs (range 4.0, 5.0, 6.0, 7.0) and their reversibility at day 0 and after 7 days of aging through a series of 3 switches between pH 7.0 and pH 5.0. Sensors surface morphology was evaluated by a scanning electron microscopy (SEM) Sigma 300VP, Zeiss, Germany, adopting as accelerating voltage of 3 kV, a secondary electron detector (SE2), and 30 000× and 100 000× magnifications. After sonication by using aDU‐32 Digital ultrasonic cl eaner, 10 µL of sensors dispersed in ethanol were deposited onto a silicon wafer and air‐dried at room temperature overnight prior SEM acquisitions. All the samples were sputter coated with a 10 nm thick gold layer in order to become conductive (Safematic CCU‐010 LV Vacuum Coating) prior to their observation under the microscope.

For SEM imaging of sensing‐integrated tumorspheres, samples were fixed in 2.5% glutaraldehyde and subsequently dehydrated through a graded ethanol series (20%, 50%, 70%, 90%, and 100%). Samples were then subjected to critical point drying (Emitech K850 CPD). The dried hydrogels were sectioned with a razor blade, mounted on aluminium stubs, and sputter‐coated with a 10 nm gold layer (Safematic CCU‐010 LV Vacuum Coating). SEM micrographs were acquired using a Sigma 300VP (Zeiss, Germany) operating at 5 kV with a secondary electron (SE2) detector.

### 4.6 Establishing Patient‐Derived Tumorspheres of PDAC

Pancreatic patient‐derived tumorspheres were generated by applying a high‐voltage microencapsulation system [23]. Briefly, pancreatic patient‐derived cells were counted by Trypan Blue dye exclusion, and 1 × 10^6^ of cells were resuspended in 50 µL of fresh medium. Then, 50 µL of alginate solution (3% of alginic acid sodium salt in distilled water) were added to the cell suspension with 10 µL of pH sensors (5.38 × 10^6^ particles/mL of the pH sensors stock solution), previously stirred for 5 min. This mix was filled into a syringe (BD Plastipak 1‐mL Syringe) and positioned into a syringe pump (World Precision Instruments, Model AL‐4000) set up to a constant flow rate of 0.05 mL min^−1^. Afterwards, a high potential difference was generated by establishing a connection between the cathode of the generator and the needle of the syringe (21 G blunt needle), whereas the anode was placed in a Petri dish holding 12 mL of CaCl_2_ solution (0.1 M CaCl_2_ and 0.4% w/v of Tween‐20) positioned at a distance of 3 cm under the syringe nozzle. Upon application of a high voltage (3.0 V), alginate‐based tumorspheres formed as the droplets fell into the calcium chloride solution. The resulting sensing‐integrated tumorspheres were rinsed three times with 10 ml of culture medium to eliminate residual CaCl_2_ solution and subsequently cultured in the incubator, before being imaged using the CLSM.

### 4.7 Determination of IC_50_

Pancreatic patient‐derived cells were counted by Trypan Blue dye exclusion and seeded in 96‐well cell culture plates (5 × 10^3^ cells/well). After 24 h, primary cells were treated with paclitaxel, FOLFIRINOX (oxaliplatin, 5‐fluorouracil, and irinotecan), and gemcitabine at doses ranging from 0 to 100 µM. PrestoBlue Cell Viability Reagent (Invitrogen, ThermoFisher Scientific) was used to test cell viability in accordance with the manufacturer's protocol. Briefly, 7 µL of PrestoBlue solution were put directly into each well and incubated for 1 h at 37°C. Then, the fluorescent signal was measured by using a plate reader (CLARIOstarplus BMG LABTECH), at 535 nm of excitation wavelength and 590 nm of emission wavelength. Each treated group was normalized with the respective control group. Lastly, the sigmoidal curves were produced by graphing the cell growth rate versus the different drug doses on a semilogarithmic scale in Graph‐Pad Prism version 8.0 software. These curves were used to determine the half maximum inhibitory concentration (IC50) values.

### 4.8 Annexin V Staining and Ki‐67 Evaluation

Apoptosis was detected using FITC‐Annexin V (Biolegend, San Diego), according to the manufacturer's protocol. Briefly, the 3D alginate tumorspheres were treated with each chemotherapeutic drug (paclitaxel, FOLFIRINOX and gemcitabine) for 12 h. The untreated tumorspheres were used as control (Basal). After treatments, patient‐derived tumorspheres were incubated with EDTA 0.5 M in order to dissolve alginate matrix and recover cells for analysis. Each sample was rinsed twice in PBS and 1 × 10^5^ cells were re‐suspended in 100 µl of Annexin V binding buffer. Then, cells were stained with 1µl of FITC‐Annexin V and Propidium iodide (PI) (20 µg/mL) for 15 min at room temperature. Then, 200 µl of Annexin V binding buffer were added in each sample before the analysis with the CytoFLEX S flow cytometer (Beckman Coulter, USA). Viable cells were defined as annexin V‐negative and PI‐negative; early apoptotic cells were defined as annexin V‐positive and PI‐negative; late apoptotic/necrotic cells were defined as annexin V‐positive and PI‐positive. In parallel, 2 × 10^5^ cells for each condition were fixed with 2% paraformaldehyde for 15 min at 4°C, incubated with saponin 0.5% and stained with PE anti‐human Ki‐67 (Biolegend Cat#350504, 1:200) for 30 min. Approximately 20.000 events were recorded for each sample and analyzed by CytExpert software. Dead cells, debris, and doublets were excluded based on forward scatter and side scatter measurements.

### 4.9 Extracellular Lactate Quantification

Changes in secreted lactate concentration in cell culture medium were measured using the Lactate Glo Assay Kit (Promega Madison, WI, USA) according to the manufacturer's protocol. Briefly, the 3D alginate tumorspheres established for each PDAC patient were treated with paclitaxel, FOLFIRINOX, and gemcitabine for 12 h. The untreated tumorspheres were used as control (Basal). Samples were collected after 1–6 and 12 h of incubation by diluting 5µL of cell culture medium into 95µL of PBS. Cell culture medium collected from each experimental time point were then analyzed through an enzymatic assay coupling lactate oxidation with NADH production detected via the bioluminescent system included in the Lactate‐Glo Assay kit. Luminescence was measured using a microplate reader (ClarioStarPlus, BMG Labtech). Medium without cells was used as a negative control for determining assay background. The data obtained were normalized by dividing the relative luminescence units (RLU) by the cell number counted for each condition. A standard curve using a titration of lactate was performed to verify that all measured samples were within the linear range indicated in the protocol from 0 to 200 µM (Data not shown). Data represent the mean ± SEM of three independent experiments (*n* = 3).

### 4.10 Live Cell Microscopy

The calibration of optical ratiometric pH sensors embedded in patient‐derived tumorspheres was performed at the beginning of each time‐lapse experiment by CLSM (Zeiss LSM 980, Carl Zeiss AG). To this purpose, samples were transferred in a 8‐well chamber slide (IBIDI, Berlin, Germany), previously treated with 0.3 mg/ml of poly‐L‐lysine. Then, patient‐derived tumorspheres were exposed to specific pH‐adjusted cell media (pHs 7.0, 6.0, 5.0, 4.0), and after 10 min of stabilization, they were acquired, along the z‐axis, by means of CLSM equipped with Okolab Stage Top Incubator (Okolab s.r.l., Italy) for regulated CO_2_ and temperature. After the calibration measurements, tumorspheres were treated with each chemotherapeutic drug and placed into the Okolab chamber. Untreated 3D alginate tumorspheres were acquired as a basal condition. The pH variations were monitored in time‐lapse mode for 12 h, and 28 z‐stack images (z‐step size: 2.55 µm) of the whole tumorspheres were recorded at an interval of 1 h; The 488 nm laser line (0.8%) was used for exciting FITC, whereas the 555 nm laser line (0.8%) was set up for exciting RBITC.

### 4.11 4D (x,y,z,t) Image Processing and Analysis

Confocal images were automatically processed with a modified version of a custom algorithm [27, 28, 29] in order to extract pH read‐outs from ratiometric florescent microsensors located around single cells immersed in a 3D alginate matrix. Briefly, for each timepoint *t*, the z‐slices of the RBITC channel were separately preprocessed, converted to grayscale, binarized, and transformed by morphological opening and closing with a disk‐shaped element to remove noise or fill holes in the objects. Then, the images were segmented by a watershed transformation [71], and the pH sensors were reconstructed in 3D by direct connectivity. The resulting matrix served as a mask to obtain positions and mean pixel‐by‐pixel ratio (*I_FITC_/I_RBITC_*) of the fluorescence intensities of the microsensors from the original FITC and RBITC images. To assign local extracellular pH values associated with individual cells, only sensors located at an Euclidean distance lower than 25 µm from the center of each cell were considered. The calibration curve obtained at the beginning of each experiment was then applied to convert *I_FITC_/I_RBITC_* values into pH measurements. The code was executed iteratively for each time point *t*, and global pH over time was monitored by calculating mean and standard deviation of the pH values recorded by individual sensors at each *t*. This procedure enabled local analysis of extracellular pH heterogeneity across the 3D tumorsphere volume.

### 4.12 Correlation of Extracellular pH Measurements and Drugs Response With Clinical Outcome

To assess the clinical relevance of the sensing‐integrated tumorspheres, extracellular pH measurements were correlated with the clinical outcomes of the corresponding PDAC patients. Local extracellular pH values were obtained from the image‐analysis pipeline by assigning to each cell the pH recorded by the surrounding ratiometric sensors after 12 h of drug treatment. For each patient and treatment condition, the distribution of cell‐associated pH values was used to classify the pH metabolic response of tumorspheres as mild (pH 7.0–6.0), moderate (pH 6.0–5.0), or severe (pH 5.0–4.0), based on extracellular acidification values previously reported for PDAC in the literature, which describe moderately acidic tumor microenvironments (pH ∼6.6) together with localized regions of more severe acidification (pH < 6.5), particularly in hypoxic tumor cores or following chemotherapy [21, 50, 61]. To qualitatively compare the predictive value of spatially resolved versus bulk pH measurements, we performed the classification using also the average extracellular pH of the entire tumorsphere. These classifications were interpreted together with the extent of drug‐induced apoptosis measured by Annexin V/PI staining after 12 h to assign each treatment as resistant (<5% increase vs Basal), moderately sensitive (>6–9% increase vs Basal), or sensitive (>10% increase vs Basal); these thresholds were empirically defined based on the distribution of Annexin V/PI‐detected cell death increase relative to basal conditions observed across all patient‐derived tumorspheres treated in this study. The resulting in vitro classification was then descriptively compared with the corresponding patient's clinical response to the administered chemotherapy, considering treatment regimen and disease‐free survival or recurrence.

### 4.13 Statistical Analysis

Data were presented as Mean ± Standard Error of the Mean (SEM). Each experiments was carried out in triplicate (*n* = 3). Statistical analyses were performed using GraphPad Prism Version 8.0. One‐way analysis of variance (ANOVA) was performed for comparison of more than two conditions, and T‐test was used for individual group comparison. p‐values < 0.05 were considered significant.

## Author Contributions

Study Conceptualization: Stefania Forciniti, Valentina Onesto, and Loretta L. del Mercato; Methodology: Stefania Forciniti, Valentina Onesto, Anna Chiara Siciliano, and Helena Iuele; Formal analysis and investigation: Stefania Forciniti, Valentina Onesto, Anna Chiara Siciliano, and Helena Iuele; Software: Valentina Onesto; Writing – original draft preparation: Stefania Forciniti. Writing – review and editing: Stefania Forciniti, Valentina Onesto, Anna Chiara Siciliano, Helena Iuele, Stefano D'Ugo, Norma Depalma, Marcello G. Spampinato, and Loretta L. del Mercato; Funding acquisition: Giuseppe Gigli and Loretta L. del Mercato; Resources: Giuseppe Gigli, Loretta L. del Mercato, Marcello G. Spampinato, Stefano D'Ugo, and Norma Depalma; Supervision: Stefania Forciniti and Loretta L. del Mercato. All authors read and approved the final paper.

## Ethics Approval and Consent to Participate

All experiments with human samples (protocol n. 32577/2023) were approved by the ASL Lecce Ethical Committee (DGR n.601/2022, DGR 683/2022 project number 13). PDAC patients enrolled in this study were from “Ospedale Vito Fazzi” (Lecce, Italy), and all procedures were conducted in accordance with the ethical principles for medical research involving human subjects.

## Conflicts of Interest

The authors declare no conflicts of interest.

## Supporting information




**Supporting File**: smll75149‐sup‐0001‐SuppMat.docx.

## Data Availability

The data that support the findings of this study are available on request from the corresponding author. The data are not publicly available due to privacy or ethical restrictions.
