# Supplementary material for: Sensing‐Integrated Patient‐Derived Tumorspheres Predict Chemotherapeutic Efficacy via Local Extracellular pH Dynamics
Source: Small. 2026 Aug 13;22(58):e75149. doi: 10.1002/smll.75149 (PMC13676843; doi:10.1002/smll.75149)
Supplement: Supplementary file 1 [file SMLL-22-e75149-s001.docx]

**Supporting information**

**Sensing-Integrated Patient-Derived Tumorspheres Predict Chemotherapeutic Efficacy via Local Extracellular pH Dynamics**

Stefania Forciniti^1,2^, Valentina Onesto^1,2^, Anna Chiara Siciliano^1,2^, Helena Iuele^1,2^, Stefano D’Ugo^3^, Norma Depalma^3^, Cinzia Fasano^4^, Alessandro D’Amuri^4,5^, Giuseppe Gigli^1,2,5^ , Marcello G. Spampinato^3,5*^, Loretta L. del Mercato^1,2*^

^1^Institute of Nanotechnology, National Research Council (CNR-NANOTEC), c/o Campus Ecotekne, via Monteroni, Lecce 73100, Italy.

^2^Tecnomed Puglia – Technopole for Precision Medicine (Biotech Lecce Hub), c/o Ecotekne Campus, via Monteroni, Lecce, 73100, Italy.

^3^Department of Surgery “Vito Fazzi” Hospital, P.zza F. Muratore 1, 73100, Lecce, Italy.

^4^UOC of Pathological Anatomy “Vito Fazzi” Hospital, P.zza F. Muratore 1, 73100, Lecce, Italy.

^5^Department of Experimental Medicine, University of Salento, c/o Campus Ecotekne, via Monteroni, 12 73100, Lecce, Italy*.*

*corresponding author: [marcello.spampinato@gmail.com](mailto:marcello.spampinato@gmail.com); [loretta.delmercato@cnr.it](mailto:loretta.delmercato@cnr.it)

**Figure S1.** Schematic summary of clinical specimens collected from “Vito Fazzi Hospital” and processed in laboratory.

**Table S1.** **Clinical and pathological characteristics of PDAC patients included in the study.**
The table summarizes demographic and tumor-related features of the patient cohort, including age, sex, histological subtype, tumor grade, and stage at diagnosis. None of the patients received neoadjuvant therapy prior to surgical resection. The primary tumor site and presence of metastatic disease are reported for each case. Cellularity (%) refers to the percentage of tumor cells present in the histological slides relative to the stromal components assessed on hematoxylin and eosin (H&E)-stained sections at 20X magnification across 10 high-power fields (HPFs). Time-to-processing indicates the interval between surgical resection and sample processing.

 **Figure S2. Flow cytometry characterization of PDAC patient-derived cells.** Cells isolated from five PDAC tumor tissues were cultured for ~20 days under specific culture conditions at 37°C and and 5% CO_2_, then fixed permeabilized, and stained with fluorescently labeled antibodies for flow cytometry analysis. **(a), (c), (e), (g), (i)** t-SNE maps showing the spatial distribution of patient-derived cells expressing mesenchymal (Vimentin, N-cadherin) and epithelial (Cytokeratin, E-cadherin and EpCAM) markers in each PDAC patients. Colored points indicate marker-positive cells, while grey points represent all the cell population in the dataset. (**b), (d), (f), (h), (j)** Quantitative distribution of epithelial markers and stromal-mesenchymal markers visualized in t-SNE maps in each patient-derived population. Percentages reflect the proportion of cells positive for each marker. Data are presented as the mean ± SEM from triplicate experiments (n=3).

**Figure S3. Flow cytometry characterization of PDAC patient-derived cells.** Histograms showing the fluorescence intensity of the characterized cells positive for the epithelial markers (Cytokeratin, E-cadherin, EpCAM,) or stromal-mesenchymal markers (Vimentin, N-cadherin,) for each PDAC patient. Isotype Control areas are shown in red, the subpopulations positive for each marker are shown in green (Vimentin), blue (Cytokeratin), cyan (E-cadherin), magenta (N-cadherin) or brown (EpCAM).

**Figure S4.** CLSM calibration curve of FITC-RBITC sensors in pH-adjusted DMEM F-12 over a wide pH range (3.5–8.5) with a superimposed Boltzmann fit. CLSM ratios were calculated as I_FITC_/I_RBITC_ (green channel: λ_EX FITC_= 488nm, λ_EM FITC_ = 500-700 nm; red channel: λ_EX RBITC_ = 543 nm, λ_EM RBITC_ = 556-700 nm).

**Figure S5.** Determination of half-maximal inhibitory concentration (IC50) on PDAC cells derived from Patient 1 **a)**, Patient 2 **b)**, Patient 3 **c)**, Patient 4 **d)**, and patient 5 **e)** at 48h after the indicated treatments by Presto Blue assay; **f)** IC50 values expressed as μM derived by the sigmoidal dose-response curves reported in **a- e)** obtained through GraphPad Prism software. Dose-response data points represent the mean value of 3 independent experiments.

**Figure S6.** Assessment of local pH changes in DMEM F-12 culture medium following drug addition. **a)** Fluorimetric calibration curve of FITC-RBITC sensors in pH-adjusted DMEM F-12 over the physiological pH range (4.0–7.0). **b)** Comparison of pH values measured in DMEM F-12 alone and after the addition of the highest concentrations of chemotherapeutic agents used in pH monitoring experiments of patient-derived tumorsphere: FOLFIRINOX (5-fluorouracil 68.7 µM, irinotecan hydrochloride 90 µM, oxaliplatin 89.1 µM), gemcitabine (35.5 µM), and paclitaxel (43.1 µM). Fluorescence measurements were performed in endpoint mode (λ_EX FITC_ = 483 ± 7 nm, λ_EM FITC_ = 530 ± 15 nm; λ_EX_ _RBITC_ = 550 ± 10 nm, λ_EM_ _RBITC_ = 605 ± 20 nm), and the emission ratio was calculated as λ_EM FITC_ / λ_EM_ _RBITC_. Data are presented as mean ± standard deviation from four independent experiments (n = 4).

**Figure S7.** Quantification of the time-lapse pH monitoring performed for each sensing-integrated patient-derived tumorsphere during drugs testing. Graphs show the mean values of the extracellular pH measurements over time extrapolated from the calibration curves obtained at the beginning of each experimental condition and plotted as function of time. Data are means ± SEM of three independent experiments (n=3).

**Figure S8.** Lactate secretion by patient-derived tumorspheres during drug testing. Alginate-based tumorspheres embedding patient-derived cells from five PDAC patients **(a-e)** were produced and treated with pharmacologically relevant concentrations of FOLFIRINOX, gemcitabine, or paclitaxel for 12 hours. At indicated time points (1-6-12 hours), 5 μl of cell culture medium was collected and diluted in 95μl of PBS and mixed with the Lactate Detection Reagent. Wells with medium only were included as controls. Luminescence signal was read using a microplate reader CLARIOstarplus BMG LABTECH. Data are presented as means ± SEM of three independent experiments (n=3).
